# A Choice Experiment Model for Honey Attributes: Italian Consumer Preferences and Socio-Demographic Profiles

**DOI:** 10.3390/nu14224797

**Published:** 2022-11-12

**Authors:** Antonina Sparacino, Valentina Maria Merlino, Simone Blanc, Danielle Borra, Stefano Massaglia

**Affiliations:** Department of Agricultural, Forest and Food Sciences, University of Turin, Largo Paolo Braccini 2, 10095 Grugliasco, Italy

**Keywords:** honey, consumer preferences, best–worst scaling, latent class analysis, consumers behaviour

## Abstract

Honey production is currently experiencing a great deal of media attention, with many positive attributes of this hive product emerging. The purpose of the study is to investigate consumer preference and what key information informs people’s purchase of honey. This study is based on consumer surveys and experimental evaluation. First of all, the relative importance assigned by consumers to 12 honey product attributes was defined by using the best–worst scaling (BWS) methodology. Secondly, the latent class analysis (LCA) was used to identify different honey consumers based on preferences. The findings demonstrate that “health aspects” and “organoleptic compound” are the main categories of information that consumers tend to research. The sample segmentation defined four different consumer clusters: people who value health, sustainability, organic sourcing and quality. Additionally, socio-demographic characteristics such as age, education level and profession also played a part on consumer choice and the characterisation of each cluster. This study can contribute to fostering good nutrition and improving sustainability within communities.

## 1. Introduction

Beekeeping is a widespread global activity, with millions of beekeepers depending on bees for their livelihoods and well-being [1]. Honey is the most well-known hive product and, since 2000, its production and consumption have been constantly increasing [2]. Honey production is currently experiencing a great deal of media attention: while in the past it was only known as a sweetener and valued for its emollient properties, today’s research highlights many different uses, which in market terms become attributes that help assess consumer behaviour.

Within the global scene, the beekeeping sector in Europe is not the biggest, but if we considered Europe as a single unit, it is the second largest producer after China. The EU countries with the largest honey production are Romania, Spain, Hungary, Germany, Italy, Greece, France and Poland, and they are mainly located in Southern Europe, where they enjoy more favourable climatic conditions for beekeeping [3]. It can be defined as a niche market in the agri-food exports’ context, even if volumes exported are increasing and in parallel to this trend consumption is also constantly increasing [4]. Beekeeping structure in Italy is peculiar because there are two types of producers: production for self-consumption conducted by hobbyist (in Italy, 71% of total production) and production specific for market (in Italy, 29% of total production) [5]. On the one hand, the presence of such many “non-professional” beekeepers is a positive value, representing a resource for the pollinator function of bees and for the ecosystem; on the other hand, not controlling production from a health point of view could be a critical issue for both the honey produced and the bees.

Honey is a special food because it contains all the nutritional elements necessary for the growth and development of organisms and human beings (amino acids, carbohydrates, vitamins, minerals, pollen, essential oils, enzymes, etc.) [3,4]. Its properties are strictly linked to health gains and therapeutic gains [4,5], thus making it a functional food [6]. Previous studies about consumer preferences investigated the influence that the communicated benefits of honey for human health have during the product purchasing process [7]. A significant portion of the current literature has examined sense-based features related to consistency (creamy, liquid or crystallised honey), taste (sweet or bitter), aroma (fruity, floral or intense) and colour [8], and how these impact consumer choice [9]. Researchers and producers are also interested in investigating gastronomic pairings, as honey plays a culinary role in preserving traditional local culture [10] (it tends to pair well with fresh or aged cheeses, meat or other ingredients in recipes).

There is great emphasis on honey beekeeping as an environmentally-friendly practice, useful to promote the local economy and to facilitate pollination in highly-valued ecosystems [8,11]. The growing sensitivity of communities towards environmental sustainability in conjunction with recent climate change has brought people (especially the younger demographics) to look to honey and bees with more interest [12]. Consumers are choosing in more environmentally-conscious ways, and think more about the sourcing and animal welfare of hive products [13,14]. On the other hand, the breeding of bees now is diffuse in all inhabited continents and this can bring to novel stressors factors like unsuitable environments and management practices or new pathogens and pests. At this purpose, considering also the increasing sensitivity of consumers towards ethical concern related to animal production, the certification of animal welfare standards can be an important driver in the consumer decision-making process.

The quality of honey is flora-related [14]; this means that every territory gives a different kind of honey in taste and properties. In addition, the link between the product quality characteristics and the territory of origin determines the uniqueness of the products of a certain territory; this uniqueness is important and aids in supporting the local economy [15] and is also positively perceived by consumers [16].

It is quite difficult for consumers to understand if one honey is better than another. During the decision-making process of choosing honey, consumers assess the quality of the product by considering different aspects of the product, such as experiential characteristics (flavour, aroma, etc.), extrinsic characteristics (such as colour or price), or credence characteristics (such as local origin or organic certification). In other cases, the concept of quality is simply related to the consumer’s loyalty to the product that is known or comes from areas close to the area of residence. The concept of quality is therefore very complex for honey, as it is for other products, where many aspects affect the quality assessment process of a product such as honey. Consequently, the adoption of diversified parameters and quality standards has resulted in an enhancement and differentiation tool that can be used by beekeepers to certify their products, as “organic” for example, in order to make a product more visible and recognisable in the eyes of the consumer as the best quality product [10]. Indeed, quality has transversal importance in the agri-food sector [17]. In Italy, only three protected denominations of origin (PDO) exist for honey (Lunigiana PDO, Dolomiti Bellunesi PDO and Varesino PDO honey), indicating that certified honey is not widespread. Much more common in the beekeeping industry are awards and competitions sponsored by various national or local associations. Therefore, honey certifications still represents a small niche market, but in the future, a growing demand for PDO honey can reasonably be expected [18]. In this sense, the role of the honey’s geographical origin, certification and territory will impact markets [19], consumer confidence in the producer [20] and purchase.

Recent studies based on consumer behaviour investigated what influences consumer choice and preference [15,19,21,22,23,24] in the agri-food sector. However, no study published to date has investigated consumer behaviour towards honey in conjunction with the changes that society, people and well-being have undergone during the COVID-19 pandemic. Moreover, the applied methodology in the present paper is widely tested and considered valid in the investigation of consumer choice behaviour [20,23,25], but it has not yet been applied to honey.

In this regard, understanding consumer preferences towards honey is important to develop marketing strategies, to increase consumer satisfaction and to boost the earnings of beekeepers. Our research hypothesis is that items and topics used as marketing strategies by beekeeping companies correspond to the needs of consumers: it is assumed that there is a match between the attributes communicated by the beekeeping companies and the honey characteristics required by consumers. In this research, we want to explore consumer preferences by addressing the following questions:

(Q1) What are the most important attributes that consumers consider before purchasing honey?

(Q2) What is the degree of importance given by the consumer to each quality attribute that describes honey?

(Q3) Is it possible to find clusters of consumers who choose in a similar way?

The methods used in this study are best–worst scaling (BWS) and latent class analysis (LCA) based on consumer surveys and experimental evaluation.

The Piedmont region in the northwest of Italy was established as the survey area. The study area was chosen given that Italy presents favourable conditions to produce different varieties of honey thanks to its mild climate and high variety of vegetation [25]. Moreover, the Italian National Institute of Services for the Agricultural Food Market (ISMEA) showed that in 2020 there were over 63,000 beekeepers (+53% compared to 2016) and over 153,000 beehives (+80% compared to 2016) with nearly 1.7 million hives. Geographically, production is widespread in all regions of the country; however, the most productive region is Piedmont, with the highest number of hives (207,339) and apiaries (21,309) [26].

## 2. Materials and Methods

### 2.1. Data Collection

A paper questionnaire was developed to explore people’s purchase and consumption preferences and habits. Four independent surveys—similar in content but different in questionnaire structure—were constructed. A choice experiment was conducted between April 2022 and August 2022 in two different ways. The first method entailed distributing the online (Google Form) questionnaire through e-mails, messaging apps, and social networks (e.g., WhatsApp, Facebook, LinkedIn). The second survey collection method consisted of face-to-face interviews (paper model) outside supermarkets by randomly selecting respondents outside stores in these geographical areas from Sunday to Saturday between 10 a.m. and 6 p.m. The survey was conducted following the ethical standards set out in the Declaration of Helsinki and approved by the Bioethics Committee of the University of Turin (n. of approval 0277021) [27]. The questionnaire excludes sensitive data and was developed in the original language of the country. It opens with a brief message introducing the general purpose of the study and explains that it is fully anonymous. Respondents were not granted any monetary incentives. To detect potential issues due to the misinterpretation of the questions posed, a pilot test was carried out with a limited number of participants (no. 10). No changes were made to the questionnaire as a result of the pilot test. In the case of the online survey, the selection of respondents was made choosing only social media communities related to the area of Piedmont region. In the case of the face-to-face interviews, the interviewer stated the research aims and invited the respondent to participate after giving consent. In both cases, the questionnaire was dedicated only to honey purchasers: in fact, an initial question was designed to ask whether the respondent corresponded to the family responsible for purchasing. Non-purchasers were discarded from the survey. Only respondents who were over 18 years of age and had completed the entire survey were selected.

The survey was structured into three main sections and its completion took 5 min on average. The first section included questions related to sociodemographic characteristics (gender, age, employment, education, number of family members and net monthly average income). The second section was created following the scheme already used in [6] and asked consumers whether they consumed honey or purchased it for other uses or other household members. The third section was devoted to the best–worst scaling methodology.

### 2.2. Best-Worst Scaling Design

The best–worst scaling (BWS) methodology is a procedural approach for collecting declared preferences among a series of attributes previously selected that describe a product; interviewees have to select the best (most important) and the worst (least important) option from a list [28]. The experimental design was performed using Sawtooth Max Diff Designer software (v.2.0.2; Sawtooth Software, Orem, UT, USA): the 12 items were combined into nine varied and different choice sets (Table 1) following the balanced incomplete block (BIB) scheme. Each set contained four attributes and a single item appeared three times in the questionnaire, as was conducted in other studies [29,30]. The software developed four different versions of the questionnaire to increase the combination of attributes. The experimental design employed in our research was already used in previous studies [28,30]. By repeatedly asking the consumer to choose the most influential/important (best) and less influential/important (worst) attribute during the selection and purchase of the product, it is possible to calculate the preference mean for each selected item. The frequency with which an attribute is selected as best (or worst) indicates the strength of the preference for that attribute [31].

In applying this methodology, 12 attributes describing characteristics, value of honey and attributes were selected from a previous study summarised in Table 2.

### 2.3. Data Analysis

Data analysis was organised into two mains stages.

The first stage focused on the assessment of the best–worst raw score for each attributes describing honey while considering the entire consumer sample. For each honey attribute, the same software used for the development of the questionnaire (Sawtooth Max Diff Designer software) was used to calculate the average raw score by deriving from the difference between the number of times each attribute was selected as worst and the number of times it was selected as best and then dividing by the sample size and the number of times each attribute appeared in the questionnaire (equal to 3 in our experimental design). To provide a more intuitive interpretation of the results, these are often rescaled into a rescaled score (0–100) where 100 is the sum of all the items [31].

The second stage of the analysis focused on identifying individuals segments with similar preferences towards honey, estimating the probability of membership to each class along with their respective class-specific preference weights [7]. Heterogeneity of preference was estimated by performing a latent class analysis (LCA). This analysis helped us to identify natural classes (clusters or segments) in the sample of respondents with homogeneous preferences [33]. LCA uses statistical criteria to test the validity of the model and generate the most appropriate numbers of cluster [34]. To achieve this, the Bayesian information criterion (BIC) value [28] and Akaike consistent information criterion (AIC) are used. To characterise the clusters and find the differences among them, authors performed analyses using IBM SPSS Statistics v.28 and applied the one-way analysis of variance (ANOVA) with post-hoc tests (Siegel–Tukey test). To strengthen cauterisation and go deeper into cluster typing, the socio-demographic characteristics of each segment was studied; a chi-square (χ^2^) was performed and the *p*-value for each question was calculated with IBM SPSS Software following a variance homogeneity test for the quantitative analysis.

## 3. Results

A total of 533 respondents participated in the survey, but only 416 (a 22% exclusion rate) were honey purchasers and, therefore, participated in the research.

Firstly, we described the results of the best–worst analysis including the general ranking of attributes and, secondly, we outlined the results obtained from the cluster analysis with sample demographic characteristics.

### 3.1. General Ranking of Attributes

The most important attributes for honey selection, with the highest average raw scores, are related to healthy benefits effects such as “Functional for the body” followed by “Strengthens immune defences” and sensorial characteristics such as “Flavour”. On the other hand, items with the lowest average raw score are “Source of sugars” and “Source of Minerals”, which relate more to nutritional characteristics, and “Certifications”, which relates to food quality (Table 3).

### 3.2. Results of the Latent Class Analysis and Description of Cluster

Following other studies [34,35] in the latent class analysis, we chose a four-class solution using the AIC and the BIC fit criteria (AIC = 16,952.252; BIC = 17,277.542). The four clusters were described according to the clustering of the variables in terms of rescaled score expressed for every single item of honey (Table 4); in the same table, the sizes of the different clusters have been shown.

From a general point of view, the distribution of the total sample within the different groups has negligible inhomogeneities. The biggest one is the “Organic is better” group (cluster 3) with 29.9% of respondents: they equate respect and care for the body with respect and care for nature: indeed, the four most important attributes for this group were “strengthens immune defences” and “is functional for the body”, “is organic” and “is environmentally sustainable”. The smallest one is the “quality-sensitive” people (cluster 4), which accounts for 20.3% of respondents. They all attach great quality value to the certification and they all pay attention to organoleptic characteristics: aroma and flavour play an important role in the honey selection decision-making process. For this group, less importance for honey choice is attributed to nutritional characteristics. People who value sustainability (cluster 2, which accounts for 23.9% of respondents) display the highest preference for environmental sustainability and animal welfare; they are passionate about the land and prefer to buy local products. For them, the role of honey goes beyond its nutritional value; it is also important for gastronomic pairing. The last group is people who consume honey for health reasons (cluster 1 with 25.8% of respondents): they prefer honey for its beneficial properties, as it strengthens immune defences and is functional for the body. They are interested in its organoleptic characteristics such as flavour and aroma. They do not care about nutritional properties or certifications.

Every single cluster was characterised based on socio-demographic characteristics that are summarised in Table 5. The differences between clusters with regard to age, education and employment are statistically significant, while the differences regarding gender, family members and monthly income are not.

## 4. Discussion

This research explored individual preferences towards different attributes of honey and dissimilar consumption profiles. The three most important attributes for consumers referred to preferences of honey were all related to human health and sensorial characteristics. Such a finding supports the notion that people consume honey based on honey benefits [21]. Honey has long been considered by the community as an effective functional food to support the prevention and treatment of diseases [5,36]; in some cases, it is used as an alternative medicine [21]. A previous study [37] found a correlation between the purchase of honey and a rich set of benefits that satisfy many needs of the human body. An important aspect identified for consumers was sensorial characteristics, especially flavour. Generally, for the purchase of food stuff, taste, aroma and flavour cover an important role because they are related to the perception and taste of individuals. The varieties of honey is a determinant for psychological factors (such as personal preferences) and social factors (family members’ tastes) [37]. However, the findings in this study do not always agree with previous literature: for example, in another study [38], consumers attributed more importance to the value, recognition and reputation of the brand, considering the honey benefits for human health less important. This difference is justified by the different attributes considered in the two studies.

In our study, nutritional characteristics and certification were deemed less important drivers of consumer preferences. With regard to certification, although it helps consumer decisions [20], the low prevalence of certification among hive products in Italy represents a gap that could be considered by producers to increase competitiveness and visibility on the market. Other studies [19,39] show that for consumers, this attribute however is not important during the honey purchasing process at local open-air market or directly at producers: in fact, the relationship of trust between producers and consumers perhaps does not require any certification. Nutritional aspects do not represent a major source of interest and we can hypothesise that the low interest related to nutritional characteristics is related to the difficulties for consumers when reading and interpreting a food label. However, cluster analysis allowed us to define heterogeneous preferences among different consumer groups. The “People who are sustainability-sensitive” group perceive honey as a product related to sustainability, animal welfare and local product concepts. More and more consumers are influenced by environmental sustainability and earth preservation as fundamental issues in their behaviour [32]. The preference of local honey also emerged in other research [15], and this is due to an increase in consumer confidence towards short supply chains [40]. According to another study [6,15], local honey is especially preferred by environmentally conscious consumers because it is seen as more environmentally benign and promotes sustainable development. Some authors find that consumers have a willingness to pay more for local food [40]. Indeed, although not statistically significant, we highlight that a higher percentage of respondents in the group than in the others have a monthly household income above 6000 EUR. The age of the respondents was statistically significant: almost half were young, between 18–29 years old, and most of them had a high level of education (42% graduates and 16% master’s degree recipients). This finding corresponds with other literature in which the environmental concerns related to food products were underlined as the most important feature for the millennial generation [23,41]. It is also statistically significant that a high percentage of students make up the cluster compared to others and most of them have a high level of education because they had graduated or had a master’s degree. Moreover, high income characterised this sustainable group in accordance with other studies in the literature [20].

The “quality-sensitive” group considered the product certification as the most important attribute for honey selection. Regarding geographical origin, a study [20] recognised certification of geographical origin as the most important attribute for Hungarian consumers; however, the general trend is to introduce a certification of the food’s preparation process and of product quality to guarantee high quality to consumers [24]. According to one researcher [18], consumers who prefer PDO honey pay attention to links to the territory of origin. Certification has a strong connection with quality and has shown a prominent role of taste in honey consumption with quality perception [9], such as aroma and flavour. Interesting to note is the characterisation of sociodemographic aspects such as age and monthly income: contrary to previous studies—where the attitude of young consumers towards certified honey is not clearly identifiable—in our study, people under the age of 44 were interested in certifications, and this is statistically significant. In addition, this is the only cluster with more males than females.

For the people who attribute more importance to “health” aspects of honey (cluster 1) and “Organic is better” (cluster 3), “health aspects” represents the most important attribute for consumers of honey. As found previously in this study and in other research [6], the consumption of hive products is linked to their effect on human health effects such as functional aspects and strengthening of immune defences. Thanks to their natural and nutritional characteristics, honey products are considered a more healthy alternative to sugar [19], and some studies [42] observed a direct correlation between honey consumption and the healthy lifestyle of consumers. The socio-demographic characteristics of the two groups taken into consideration differ in age and employment: like older people, young people are interested in healthy food, but in a different way. For the first category (i.e., people who attribute importance to health) aroma, taste, flavour and all organoleptic compounds play a fundamental role in terms of choice, supported especially by young respondents [9]. While for the “Organic is better” category, which is mostly made up of adults and retirees, what is statistically significant is the organic nature of honey, which contributes to their well-being and helps in preventive health action [43]. Their diets are characterised mainly by fresh fruit and vegetables [44], while interest in organic food can drive good opportunities for environmentally sustainable beekeeping [15]. Lastly, what is also interesting is that the gender composition of the group “Organic is better”: according to some studies, women consumed honey more frequently and had higher levels of health and nutrition awareness [19].

This research contributes to the literature by improving the knowledge of honey consumers and has several implications for academics and beekeepers. The findings can contribute to promoting good nutrition and improving public health. In particular, given that the study was conducted in the most productive region of Italy at national level, but also in the second most important area (Northwest) in terms of honey purchases [43], these results have an important value for the whole national production sector. Furthermore, the experimental model used in the study could be replicated in other consumption areas, nationally and internationally, to make comparisons between honey consumption styles and preferences. Thus, while the limited study area may be a limitation of this study, it represents a good starting point for future research carried out with a view to exploring perspectives of the honey consumer on a broad spectrum by comparing different geographical areas.

Another limitation of this study could be identified in the attributes selected for the analysis. Extrinsic attributes, such as branding, pricing and labelling, were not included; only intrinsic attributes and credence were looked at to obtain the results. However, this latter limitation could be used to improve the experimental design by using additional attributes in future studies.

## 5. Conclusions

In the agri-food sector, consumer choice often has to do with flavour and taste. The results of this study confirm the findings of other researchers: attention to the human body and health aspects, interest in nature and the short supply chain and media attention for environment sustainability have changed the ways we consume and buy food. This study confirms that honey consumer behaviours are increasingly linked to health aspects and organoleptic compounds. Moreover, it was possible to identify different types of consumers based on their preferences: healthy people, sustainable people, organic people and quality-sensitive people. Additionally, some socio-demographic characteristics such as age, education level and profession influenced consumer choice and the characterisation of each cluster. Interest towards sustainability issues characterises young people; instead, health benefits represent the most important aspect for young people and adults. Therefore, also in case of honey, the preferences definitions are affected by individual profiles and characteristics.

## Figures and Tables

**Table 1:** Example of best–worst question.

Most Important (Best)	Attributes	Least Important (Worst)
-	Gastronomic pairing	-
-	Link to the territory	-
-	Certification (of process, origin)	-
-	Organic	-

**Table 2:** Description of 12 attributes used to identification honey preferences.

Categories	Sub-Categories	Description	References
Health aspects	Strengthens immune defences	Naturally antibiotic, antibacterial and antiviral functions; it strengthens the immune defences.	[6,7,12]
	Functional for the body	It regulates kidney function; it enriches the intestinal flora and promotes skin elasticity. It has antiseptic properties and in general is good for the body.	
Nutritional characteristics			
	Source of minerals	Contains mineral salts.	
	Source of sugars	Source of simple sugars, such as glucose and fructose, and complex sugars.	
Origin			
	Link with the territory	The territory where honey is produced thanks to local flora. The respect, protection and promotion of local sources are included in this attribute.	[10,19,29,32]
	Gastronomic pairing	The combination of honey with other foods. Honey in the Italian gastronomic culture is associated with other traditional products of the land, i.e., cheeses and cured meats. As these are gastronomic combinations, the use of honey as a sweetener, e. g., in tea and herbal teas, is excluded from this codification.	
Quality	Quality and certifications	In this case, quality is meant as good quality, high quality, and excellent quality mainly referred to the product in a generic way. It also includes the presence of other certifications (excuses organic), bee industry awards, production certifications such as ISO 22000 and international certifications such as the International Food Standard (IFS).	[18,25,29,31]
	Organic	Organic production often represents an added value. If present, the organic certification logo or procedure is usually communicated on the website.	
Sustainability	Animal welfare	Referring to certification about welfare and safekeeping of bees to guarantee animal protection and respecting their ecosystem.	[11,17,33]
	Environmental sustainability	Safeguarding the environment and biodiversity.	
Sensorial characteristics	flavour	Taste.	[8,9,22]
	Aroma	Scent and aroma.	

**Table 3:** Ranking and aggregate average importance score, and the number of times honey attributes were selected as best or worst.

Label	Times Selected (Best)	Times Selected (Worst)	Average Raw Score	Standard Deviation
Functional for the body	556	106	1.865	1.619
Strengthens immune defences	501	189	1.249	2.093
Flavour	437	214	1.142	1.823
Environmental sustainability	389	152	0.861	1.406
Links with the territory	381	299	0.502	1.823
Organic	306	227	0.292	1.789
Aroma	297	309	0.002	1.659
Animal welfare	237	230	−0.066	1.627
Gastronomic pairing	251	314	−0.169	1.734
Source of minerals	113	508	−1.586	1.563
Certifications	183	592	−1.890	2.416
Source of sugars	93	604	−2.203	1.456

**Table 4:** Characterisation of clusters based on segmentation variables (probability scale: 0–100 Rescaled Score values).

	People Who Value Health in Honey	People Who are Sustainability-Sensitive	People Who Think Organic is Better	People Who are Quality-Sensitive	F	Sig.
Segment Sizes	25.8%	23.9%	29.9%	20.4%		
Gastronomic pairing	3.581 ^a^	12.514 ^c^	6.709 ^b^	5.189 ^a,b^	45.104	***
Link with the territory	4.467 ^a^	14.276 ^c^	9.148 ^b^	8.659 ^b^	31.146	***
Certifications	2.078 ^a^	1.673 ^a^	1.126 ^a^	15.797 ^b^	199.008	***
Organic	6.267 ^a^	9.750 ^b^	10.441 ^b^	5.819 ^a^	12.048	***
Aroma	11.695 ^c^	6.202 ^b^	2.668 ^a^	12.542 ^d^	115.661	***
Flavour	18.281 ^c^	10.836 ^b^	4.261 ^a^	16.980 ^c^	149.519	***
Source of sugars	2.184 ^a,b^	1.542 ^a^	2.595 ^b^	3.296 ^b^	5.284	***
Source of minerals	3.237 ^b^	1.634 ^a^	5.180 ^c^	2.785 ^a^	22.053	***
Environmental sustainability	6.127 ^a^	16.816 ^c^	11.908 ^b^	7.655 ^a^	104.701	***
Animal welfare	3.8669 ^a^	13.831 ^c^	6.822 ^b^	4.727 ^a^	63.767	***
Strengthens immune defences	17.617 ^c^	3.819 ^a^	19.083 ^c^	7.475 ^b^	217.209	***
Functional for the body	20.594 ^c^	7.100 ^a^	20.053 ^c^	9.069 ^b^	250.469	***

^a,b,c,d^ The preference averages (rescaled scores) within a row with the same letters are statistically different (α = 0.05, Tukey’s post-hoc test). Significance level: *p*-value < 0.001 ***.

**Table 5:** Description of the sample and cluster characterisation.

	People Who Value Health in Honey	People Who are Sustainability-Sensitive	People Who Think Organic is Better	People Who are Quality-Sensitive	Total	χ^2^	*p*-Value
Segment Sizes	25.8%	23.9%	29.9%	20.4%			
Female	58%	58%	61%	46%	57%	6.191	0.103
Male	42%	42%	38%	54%	43%		
18–29	30%	47%	12%	39%	30%	48.632	***
30–44	19%	16%	25%	31%	22%		
45–59	35%	28%	39%	19%	32%		
60 & over	16%	9%	25%	11%	16%		
Primary School	1%	0%	0%	0%	0%	23.760	**
Middle School	8%	6%	15%	9%	10%		
High School	43%	36%	45%	41%	41%		
Graduate	42%	42%	36%	47%	41%		
Master	6%	16%	4%	3%	7%		
Employed	64%	56%	51%	60%	57%	29.434	**
Self-employed	9%	11%	17%	12%	13%		
Homemaker	2%	3%	8%	0%	4%		
Unemployed	5%	5%	2%	4%	4%		
Student	9%	19%	7%	16%	12%		
Retired	10%	5%	15%	7%	10%		
1 member	14%	13%	8%	11%	11%	10.810	0.545
2 members	24%	24%	36%	30%	29%		
3 members	27%	26%	25%	19%	25%		
4 members	25%	26%	25%	30%	26%		
5 or more members	10%	11%	7%	10%	9%		
Less than 1.000 EUR	4%	4%	2%	3%	3%	17.393	0.296
1.000–2.000 EUR	22%	24%	28%	16%	23%		
2.000–4.000 EUR	33%	31%	33%	49%	36%		
4.000–6.000 EUR	17%	10%	14%	7%	12%		
More than 6.000 EUR	6%	14%	4%	7%	8%		
No answer	4%	4%	6%	4%	18%		

Significance level: *p*-value < 0.05 **; < 0.001 ***.

## Data Availability

Not applicable.

## References

[1] FAO (2019). Honey.

[2] Beehive Numbers Worldwide 2010–2020. Statista n.d. https://www.statista.com/statistics/818286/number-of-beehives-worldwide/.

[3] Ćirić M., Ignjatijević S., Cvijanović D. (2015). Research of Honey Consumers’ Behavior in Province of Vojvodina. Econ. Agric..

[4] Cianciosi D., Forbes-Hernández T.Y., Afrin S., Gasparrini M., Reboredo-Rodriguez P., Manna P.P., Zhang J., Lamas L.B., Florez S.M., Toyos P.A. (2018). Phenolic Compounds in Honey and Their Associated Health Benefits: A Review. Molecules.

[5] Arawwawala M., Hewageegana S. (2017). Health Benefits and Traditional Uses of Honey: A Review. J. Apitherapy.

[6] Zanchini R., Blanc S., Pippinato L., Di Vita G., Brun F. (2022). Consumers’ attitude towards honey consumption for its health benefits: First insights from an econometric approach. Br. Food J..

[7] Viscecchia R., Nocella G., De Devitiis B., Bimbo F., Carlucci D., Seccia A., Nardone G. (2019). Consumers’ trade-off between nutrition and health claims under regulation 1924/2006: Insights from a choice experiment analysis. Nutrients.

[8] Ghorab A., Rodríguez-Flores M.S., Nakib R., Escuredo O., Haderbache L., Bekdouche F., Seijo M.C. (2021). Sensorial, Melissopalynological and Physico-Chemical Characteristics of Honey from Babors Kabylia’s Region (Algeria). Foods.

[9] Šedí k P., Kňazovická V., Horská E., Kačániová M. (2018). Consumer sensory evaluation of honey across age cohorts in Slovakia. Potravin. Slovak. J. Food Sci..

[10] Zhang T., Chen J., Hu B. (2019). Authenticity, Quality, and Loyalty: Local Food and Sustainable Tourism Experience. Sustainability.

[11] Pippinato L., Blanc S., Mancuso T., Brun F. (2020). A Sustainable Niche Market: How Does Honey Behave?. Sustainability.

[12] Sillman J., Uusitalo V., Tapanen T., Salonen A., Soukka R., Kahiluoto H. (2021). Contribution of honeybees towards the net environmental benefits of food. Sci. Total Environ..

[13] Blanc S., Accastello C., Girgenti V., Brun F., Mosso A. (2018). Innovative Strategies for the Raspberry Supply Chain: An Environmental and Economic Assessment. Food Saf. Manag..

[14] Fontana P., Costa C., Di Prisco G., Ruzzier E., Annoscia D., Battisti A., Caoduro G., Carpana C., Contessi A., Dal Lago A. (2018). Appeal for biodiversity protection of native honey bee subspecies of apis mellifera in Italy (San michele all’Adige declaration). Bull. Insectology.

[15] Cosmina M., Gallenti G., Marangon F., Troiano S. (2016). Reprint of “Attitudes towards honey among Italian consumers: A choice experiment approach”. Appetite.

[16] Sundbo D.C. (2013). Local food: The social construction of a concept. Acta Agric. Scand. Sect. B Soil Plant Sci..

[17] Qaim M. (2017). Globalisation of agrifood systems and sustainable nutrition. Proc. Nutr. Soc..

[18] Di Vita G., Pippinato L., Blanc S., Zanchini R., Mosso A., Brun F. (2021). Understanding the Role of Purchasing Predictors in the Consumer’s Preferences for PDO Labelled Honey. J. Food Prod. Mark..

[19] Kowalczuk I., Jeżewska-Zychowicz M., Trafiałek J. (2017). Conditions of Honey Consumption in Selected Regions of Poland. Acta Sci. Pol. Technol. Aliment..

[20] Oravecz T., Mucha L., Magda R., Totth G., Illés C. (2020). Consumers’ Preferences for Locally Produced Honey in Hungary. Acta Univ. Agric. Silvic. Mendel. Brun..

[21] Purnomo D., Bunyamin A., Gunawan W., Faizah N.A., Danuwidjaja T.G., Rohman L.N., Annisa R. (2021). Motivation, purpose, and purchasing frequency of honey consumption in West Java. IOP Conf. Ser. Earth Environ. Sci..

[22] Selmi S., Irnad I., Sistanto S. (2020). Segmentation of consumers of honey and identification of honey preference in Kota Bengkulu. Agritropica J. Agric. Sci..

[23] Blanc S., Zanchini R., Di Vita G., Brun F. (2021). The role of intrinsic and extrinsic characteristics of honey for Italian millennial consumers. Br. Food J..

[24] Brščić K., Šugar T., Poljuha D. (2017). An empirical examination of consumer preferences for honey in Croatia. Appl. Econ..

[25] Nandi R., Bokelmanna W., Gowdru N., de Souza Dias G.H. (2014). Consumer Preferences and Influencing Factors for Purchase Places of Organic Food Products: Empirical Evidence from South India. Indian J. Mark..

[26] M- Api e miele—News e analisi—Tendenze—n.1/2020. https://www.ismeamercati.it/flex/cm/pages/ServeBLOB.php/L/IT/IDPagina/11223.

[27] (2014). Comitato di Bioetica dell’Ateneo. https://www.unito.it/ricerca/strutture-e-organi-la-ricerca/comitato-di-bioetica-dellateneo.

[28] Massaglia S., Borra D., Peano C., Sottile F., Merlino V.M. (2019). Consumer Preference Heterogeneity Evaluation in Fruit and Vegetable Purchasing Decisions Using the Best–Worst Approach. Foods.

[29] Goodman S., Lockshin L., Cohen E. Best-Worst Scaling: A simple method to determine drinks and wine style preferences. Proceedings of the 2nd International Wine Marketing Symposium.

[30] Merlino V.M., Borra D., Girgenti V., Dal Vecchio A., Massaglia S. (2018). Beef meat preferences of consumers from Northwest Italy: Analysis of choice attributes. Meat Sci..

[31] Madureira H., Nunes F., Oliveira J.V., Madureira T. (2018). Preferences for Urban Green Space Characteristics: A Comparative Study in Three Portuguese Cities. Environments.

[32] Blanc S., Brun F. (2019). Traditional Beekeeping in Rural Areas: Profitability Analysis and Feasibility of Pollination Service. Calitatea.

[33] Li Q., Rezaei J., Tavasszy L., Wiegmans B., Guo J., Tang Y., Peng Q. (2020). Customers’ preferences for freight service attributes of China Railway Express. Transp. Res. Part Policy Pract..

[34] Linder M.O., Sidali K.L., Fischer C., Gauly M., Busch G. (2022). Assessing Italians’ Preferences for Mountain Beef Production Using a Best–Worst Scaling Approach. Mt. Res. Dev..

[35] Boekel L.C., van Peek S.T., Luijkx K.G. (2017). Diversity in Older Adults’ Use of the Internet: Identifying Subgroups Through Latent Class Analysis. J. Med. Internet Res..

[36] Kumar K.P.S., Bhowmik D., Chandira M.R. (2010). Medicinal uses and health benefits of Honey: An Overview. J. Chem. Pharm. Res..

[37] Roman A., Popiela-Pleban E., Kozak M., Roman K. (2013). Factors influencing consumer behavior relating to the purchase of honey part 2. Product quality and packaging. J. Apic. Sci..

[38] Batt P.J., Liu A. (2012). Consumer behaviour towards honey products in Western Australia. Br. Food J..

[39] Pocol C.B., Bolboacă S.D. (2013). Perceptions and trends related to the consumption of honey: A case study of North-West Romania. Int. J. Consum. Stud..

[40] Wu S., Fooks J.R., Messer K.D., Delaney D. (2015). Consumer demand for local honey. Appl. Econ..

[41] Eldesouky A., Mesias F.J., Escribano M. (2020). Consumer Assessment of Sustainability Traits in Meat Production. A Choice Experiment Study in Spain. Sustainability.

[42] Pocol C.B., Šedík P., Horská E. (2018). Honey Consumption Patterns of Young People in Romania. International Scientific Days 2018: Towards Productive, Sustainable and Resilient Global Agriculture and Food Systems Proceedings.

[43] Makatouni A. (2002). What motivates consumers to buy organic food in the UK? Results from a qualitative study. Br. Food J..

[44] Savelli E., Murmura F., Liberatore L., Casolani N., Bravi L. (2019). Consumer attitude and behaviour towards food quality among the young ones: Empirical evidences from a survey. Total Qual. Manag. Bus Excell..

